# Identifying delayed left ventricular lateral wall activation in patients with non-specific intraventricular conduction delay using coronary venous electroanatomical mapping

**DOI:** 10.1007/s12471-015-0777-3

**Published:** 2015-12-03

**Authors:** A.M.W. van Stipdonk, M. Mafi Rad, J.G.L.M. Luermans, H.J. Crijns, F.W. Prinzen, K. Vernooy

**Affiliations:** 1 Department of Cardiology, Maastricht University Medical Center, PO Box 5800, 6202AZ Maastricht, The Netherlands; 2 Department of Physiology, Cardiovascular Research Institute Maastricht (CARIM), Maastricht University, Maastricht, The Netherlands

**Keywords:** Cardiac resynchronisation therapy, Coronary venous electroanatomical mapping, Non-specific intraventricular conduction delay, QRS characteristics

## Abstract

**Background:**

Delayed left ventricular (LV) lateral wall activation is considered the electrical substrate that characterises patients suitable for cardiac resynchronisation therapy (CRT). Although typically associated with left bundle branch block, delayed LV lateral wall activation may also be present in patients with non-specific intraventricular conduction delay (IVCD). We assessed LV lateral wall activation in a cohort of CRT candidates with IVCD using coronary venous electroanatomical mapping, and investigated whether baseline QRS characteristics on the ECG can identify delayed LV lateral wall activation in this group of patients.

**Methods:**

Twenty-three consecutive CRT candidates with IVCD underwent intra-procedural coronary venous electroanatomical mapping using EnSite NavX. Electrical activation time was measured in milliseconds from QRS onset and expressed as percentage of QRS duration. LV lateral wall activation was considered delayed if maximal activation time measured at the LV lateral wall (LVLW-AT) exceeded 75 % of the QRS duration. QRS morphology, duration, fragmentation, axis deviation, and left anterior/posterior fascicular block were assessed on baseline ECGs.

**Results:**

Delayed LV lateral wall activation occurred in 12/23 patients (maximal LVLW-AT = 133 ± 20 ms [83 ± 5 % of QRS duration]). In these patients, the latest activated region was consistently located on the basal lateral wall. QRS duration, and prevalence of QRS fragmentation and left/right axis deviation, and left anterior/posterior fascicular block did not differ between patients with and without delayed LV lateral wall activation.

**Conclusion:**

Coronary venous electroanatomical mapping can be used at the time of CRT implantation to determine the presence of delayed LV lateral wall activation in patients with IVCD. QRS characteristics on the ECG seem unable to identify delayed LV lateral wall activation in this subgroup of patients.

## Background

The supposed mechanism of the benefit of cardiac resynchronisation therapy (CRT) is that delayed activation of the left ventricular (LV) lateral wall causes mechanical dysfunction, which can be reverted by paced pre-excitation of this delayed LV region. Left bundle branch block (LBBB) is typically associated with early septal activation and delayed activation of the LV lateral wall [1–3]. Non-specific intraventricular conduction delay (IVCD), on the other hand, is considered a more heterogeneous group of conduction disorders exhibiting a more variable pattern of ventricular electrical activation [3]. This may explain why, in contrast to patients with LBBB, patients with IVCD show a variable response to CRT [4]. The reduced benefit of CRT observed in this subgroup of patients has led international guidelines to assign a lower level of recommendation to CRT in patients with IVCD [5]. However, recent studies have suggested that CRT may be beneficial in a subset of IVCD patients with evidence of LV activation delay [6–9]. Recently, we introduced coronary venous electroanatomical mapping as a tool to assess LV electrical activation at the time of CRT implantation in patients with LBBB [10].

The objectives of the present study were [1] to investigate whether coronary venous electroanatomical mapping can be used at the time of CRT implantation to determine the presence of delayed LV lateral wall activation in patients with IVCD, and [2] to investigate whether QRS characteristics on the ECG, other than QRS morphology, can identify delayed LV lateral wall activation as determined by coronary venous electroanatomical mapping in this subgroup patients.

## Methods

### Study population

Twenty-three consecutive patients referred for CRT device implantation, with LV ejection fraction (LVEF) < 35 %, New York Heart Association (NYHA) functional class II, III or ambulatory IV, and IVCD with QRS duration ≥ 120 ms were enrolled. IVCD was defined as a QRS duration ≥ 120 ms without typical features of LBBB or RBBB, according to accepted criteria [11]. The study protocol was approved by the Institutional Review Board.

### Electroanatomical mapping

Coronary venous 3D electroanatomical mapping was performed at the time of CRT implantation as described previously [10]. In brief, prior to LV lead placement, a 0.014 inch guidewire (Vision Wire, Biotronik SE & Co.KG), which permits unipolar sensing and pacing, was inserted into the coronary sinus and connected to an EnSite NavX system (St Jude Medical, St Paul, MN, USA). The guidewire was manipulated to all coronary sinus branches located on the inferolateral or anterolateral LV wall as defined by the American Heart Association (AHA) 17-segment heart model [12], creating an anatomic map along with determining local electrical activation time during intrinsic ventricular activation. Local activation time was measured in milliseconds (ms) from QRS onset on surface ECG and expressed as percentage of total QRS duration. Activation of the LV lateral wall was considered delayed if maximal activation time measured at the LV lateral wall (maximal LVLW-AT) exceeded 75 % of the total QRS duration. This definition was chosen because epicardial mapping via the coronary veins is limited by coronary venous anatomy, which means that some areas cannot be mapped because they do not contain any veins. Therefore, the latest activated LV region can only be identified using coronary venous mapping by relating the electrical activation time of the anatomical region to its time point within the QRS complex. We believe that an electrical activation time exceeding 75 % of QRS duration is a reasonable definition for delayed LV lateral wall activation, especially since previous studies have shown that positioning of the LV lead over a region of the heart with an electrical delay of just over 50 % of the total QRS duration is associated with a superior CRT outcome [9, 13].

After the mapping procedure, the LV lead was positioned in or as close as possible to the region of maximal electrical delay based on current evidence and recommendations [14, 15].

### ECG assessment

Twelve-lead ECGs were assessed by two experienced clinicians blinded to patient data and mapping results. Any disagreement was reviewed together before achieving consensus. QRS duration was assessed in the lead with the widest QRS. The QRS axis was derived from the automatically computed value on the ECG. Left and right axis deviation, as well as the presence of left anterior and posterior fascicular block, were defined according to AHA/ACC/HRS criteria [11], excluding the criterion of QRS duration < 120 ms because of coexisting IVCD. QRS fragmentation was defined according to Das et al. [16]; > 2 notches in at least 2 contiguous leads, or multiple notches in the R wave, or > 2 notches in the nadir of the S wave. The region of notching was classified as anterior when observed in leads V1–5, inferior when observed in II, III, aVF and lateral when observed in leads I, aVL, V6.

### Statistical analysis

Continuous variables are expressed as mean ± standard deviation and were compared using the Mann-Whitney U test. Categorical variables are expressed as observed numbers and percentage values, and were compared using Fisher’s exact test. Statistical significance was accepted at the 95 % confidence interval (*p* < 0.05). Statistical analysis was performed using SPSS version 22.0 (SPSS Inc.) software.

## Results

### Patient characteristics

Twenty-three patients with IVCD referred for CRT implantation were included in this study. The patient characteristics are presented in Table 1.

**Table 1:** Patient characteristics (*n* = 23)

Variable	Value
Men	16 (64)
Age	72 ± 10
Ischaemic aetiology	18 (72)
NYHA class	
II	15 (60)
III	9 (36)
IV	1 (4)
LVEF (%)	29 ± 5
QRS duration (ms)	158 ± 23

Values are in means ± standard deviations or numbers (percentage values).

*NYHA* New York Heart Association, *LVEF* left ventricular ejection fraction.

### Results of electroanatomical mapping

Coronary venous electroanatomical mapping was accomplished without complications in all patients. A mean number of 2.8 ± 0.7 coronary sinus branches were mapped during the procedure, of which 1.9 ± 0.5 were located on the LV lateral wall. Three-dimensional electrical activation maps were generated from 79 ± 18 unique anatomic points. Mapping time was 18 ± 5 min, fluoroscopy time during the entire procedure 19 ± 4 min, and total radiation dosage 4031 ± 2064 cGy × cm^2^.

Delayed LV lateral wall activation, defined as maximal LVLW-AT exceeding 75 % of the total QRS duration, occurred in 12/23 (52 %) patients. In Table 2, LVLW-AT data are presented for patients with and without delayed LV lateral wall activation. In patients with delayed LV lateral wall activation, maximal LVLW-AT was 133 ± 20 ms (83 ± 5 % of QRS duration) and ranged from 103 to 181 ms (75–93 % of QRS duration). In patients without delayed LV lateral wall activation, maximal LVLW-AT was 100 ± 19 ms (64 ± 9 % of QRS duration) and ranged from 69 to 138 ms (45–74 % of QRS duration). The number of lateral veins that were mapped did not differ between patients with and without delayed LV lateral wall activation (*p* = 0.92). Also, baseline characteristics did not differ between the two groups. In patients with delayed LV lateral wall activation, the most delayed lateral region was more frequently located on the ‘basal’ lateral wall than in patients without delayed LV lateral wall activation (*p* = 0.03).

**Table 2:** LV lateral wall activation time and latest activated regions in patients with and without delayed LV lateral wall activation

Variable	Delayed LV activation (*n* = 12)	No delayed LV activation (*n* = 11)	*P*
Maximal LVLW-AT (ms)	133 (± 20)	100 (± 19)	
Maximal LVLW-AT (% of QRS)	83 (± 5)	64 (± 9)	
Region of maximal LVLW-AT			
Basal anterolateral	7 (58.3)	2 (18.2)	0.09
Basal inferolateral	4 (33.3)	3 (27.3)	1.00
Mid anterolateral	1(8.3)	4 (36.4)	0.16
Mid inferolateral	0	2 (18.2)	0.22

Values are in means ± standard deviations or numbers (percentage values).

*LVLW-AT* left ventricular lateral wall activation time.

**Fig. 1:**
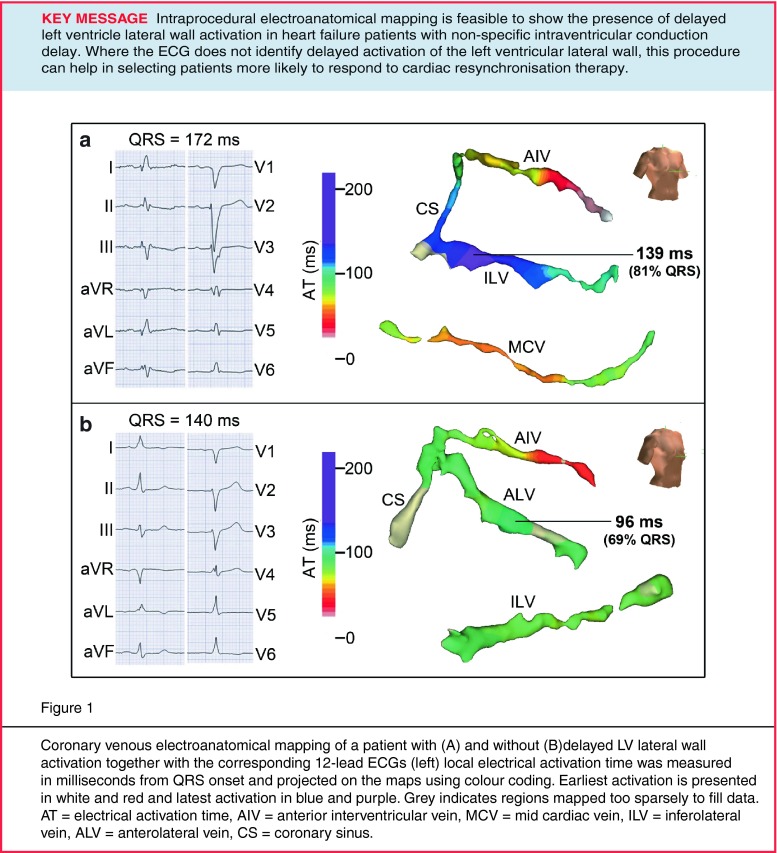
Coronary venous electroanatomical mapping of a patient with (**a**) and without (**b**) delayed LV lateral wall activation together with the corresponding 12-lead ECGs (*left*). Local electrical activation time was measured in milliseconds from QRS onset and projected on the maps using colour coding. Earliest activation is presented in *white* and *red* and latest activation in *blue* and *purple*. *Grey* indicates regions mapped too sparsely to fill data. *AT* electrical activation time, *AIV* anterior interventricular vein, *MCV* mid cardiac vein, *ILV* inferolateral vein, *ALV* anterolateral vein, *CS* coronary sinus

Figure 1 shows examples of coronary venous electroanatomical mapping of 2 patients with and without delayed LV lateral wall activation. In the example shown in panel A, the coronary sinus and three side branches located on the anterior, inferior and inferolateral wall were mapped. Delayed activation (139 ms [80 % of QRS duration]) was found in the basal region of the inferolateral vein. In the example shown in panel B, coronary veins on both the anterolateral and inferolateral wall were mapped, but delayed activation could not be measured in either vein.

### ECG parameters in patients with and without delayed LV lateral wall activation

The ECG characteristics of patients with and without delayed LV lateral wall activation are presented in Table 3. QRS duration did not differ between patients with and without delayed LV lateral wall activation. Neither was there a difference in the prevalence of left axis deviation, right axis deviation, left anterior fascicular block, left posterior fascicular block or QRS fragmentation between patients with and without delayed LV lateral wall activation. There was also no difference in the distribution of the regions with QRS fragmentation between the two groups.

**Table 3:** ECG parameters in patients with and without delayed activation of the LV lateral wall

Variable	Delayed LVLW activation (*n* = 12)	No delayed LVLW activation (*n* = 11)	*P*
QRS duration (ms)	159 (± 19)	157 (± 27)	0.46
Left axis deviation	5 (46)	6 (55)	1.00
Right axis deviation	1 (9)	0	1.00
LAFB	4 (36)	6 (55)	0.67
LPFB	1 (9)	0	1.00
QRS fragmentation	7 (64)	5 (46)	0.67
Region of QRS fragmentation			
Anterior	2 (18)	2 (17)	1.00
Inferior	4 (33)	3 (27)	1.00
Lateral	5 (42)	2 (18)	0.37

Values are in means ± standard deviations or numbers (percentage values).

*LVLW* left ventricular lateral wall, *LAFB* left anterior fascicular block, *LPFB* left posterior fascicular block.

## Discussion

Delayed activation of the LV lateral wall is considered the electrical substrate underlying LV dysfunction amenable to CRT, and may be present in patients without LBBB QRS morphology [6, 17]. The present study demonstrates that coronary venous electroanatomical mapping can be used at the time of CRT implantation to determine the presence of delayed LV lateral wall activation and specify the latest activated region in CRT candidates with IVCD. Delayed LV lateral wall activation was found in approximately half of the patients. In patients with delayed activation, the location of the latest activated region was practically confined to the ‘basal’ lateral wall, whereas patients without delayed activation showed a more heterogeneous distribution of the latest activated region on the LV lateral wall. Baseline 12-lead ECG QRS characteristics were not able to identify the presence of delayed LV lateral wall activation as determined by coronary venous electroanatomical mapping.

### Delayed LV lateral wall activation in IVCD patients

The present study demonstrates that coronary venous electroanatomical mapping can be used at the time of CRT implantation to identify IVCD patients with delayed LV lateral wall activation.

Detailed 3D LV mapping in patients with IVCD has so far been limited to a single case series recently published by Ploux et al. [6]. The authors characterised LV epicardial electrical activation of 15 IVCD patients referred for CRT implantation using non-invasive 3D electrocardiographic imaging and found significant LV activation delay in 3 (20 %) of these patients. Additionally, the presence of LV activation delay in IVCD patients was shown to be associated with clinical response to CRT. Several other studies have also suggested that CRT may be beneficial in a subset of non-LBBB patients with evidence of LV activation delay [9, 18, 19].

### Latest activated region

In patients with delayed LV lateral wall activation, the region of latest activation was similar to that observed in previous mapping studies of patients with LBBB [2, 3, 6, 20]. This observation suggests the presence of a conduction disturbance at a similar level of that observed in patients with LBBB despite the absence of a typical LBBB morphology on surface ECG. On the other hand, in patients without delayed LV lateral wall activation, the location of the latest activated region was more heterogeneous, which suggests that QRS widening in these patients may have a different underlying aetiology such as LV hypertrophy, LV enlargement, myocardial fibrosis or conduction disturbances located more distally than the usual sites at the bundle branches [3]. These findings correspond with the results of a recent study by Eschalier et al. who characterised a subset of patients with IVCD that had ventricular conduction properties similar to that of LBBB patients, but did not display typical LBBB characteristics on the 12-lead ECG [21].

### Diagnostic performance of ECG parameters for delayed LV lateral wall activation

Patient selection in CRT has been primarily based on a wide QRS complex [5]. Yet in the present study we found that a larger QRS duration was not associated with delayed LV lateral wall activation in patients with IVCD. This may be explained by the fact that QRS duration is merely a surface depiction of biventricular depolarisation time, sensitive to conduction delay in either the left or right ventricle.

With respect to the frontal QRS axis, neither left nor right axis deviation was associated with delayed LV lateral wall activation in the present study. Especially the lack of delayed LV lateral wall activation in patients with left axis deviation was remarkable, as this is considered a sign of LV involvement in conduction delay. A possible explanation for this finding is that in heart failure patients, axis deviation may often be the result of a change in the anatomical position of the heart due to for instance LV dilatation, rather than an actual change in the vector of electrical conduction [22, 23]. Although even more specific for left ventricular conduction delay, in this study neither anterior nor posterior fascicular block was associated with the presence of delayed LV lateral wall activation.

Given the fact that the presence of myocardial scar and ischaemic cardiomyopathy in a more broader sense have been shown to reduce the likelihood of a favourable response to CRT [24–26], we hypothesised that ECG markers of myocardial scarring/fibrosis have an inverse relationship with delayed LV lateral wall activation. Various ECG parameters have been related to the presence or extent of myocardial scarring [27]. Yet the majority of these parameters have not been validated in patients with ventricular conduction disorders. QRS fragmentation has previously been shown to indicate the presence of myocardial scarring and to predict worse outcome in patients with conduction disorders [16, 28]. However, in the present study a significant relationship between QRS fragmentation and delayed LV lateral wall was not found. These results are in line with the findings of two earlier studies that found no relation between the presence of QRS fragmentation and echocardiographic response to CRT [29, 30].

### Limitations

The present study demonstrates the presence of delayed LV lateral wall activation in approximately 50 % of IVCD patients. Admittedly, the study lacks data on acute haemodynamic response and long-term clinical outcome. Whether delayed LV lateral wall activation is also associated with CRT response in this group of patients needs to be confirmed in subsequent, larger and long-term follow-up studies. The small sample size of this study precludes extrapolating the results in our cohort of IVCD patients to larger populations. Nevertheless, the study population was large enough to demonstrate the feasibility of coronary venous electroanatomical mapping to determine the presence of delayed LV lateral wall activation in CRT candidates with IVCD.

## Conclusion

Coronary venous electroanatomical mapping can be used at the time of CRT implantation to identify IVCD patients with delayed LV lateral wall activation, who are more likely to benefit from CRT. Baseline QRS characteristics on the ECG seem unable to identify delayed LV lateral wall activation in this subgroup of patients.

### Disclosures

Frits Prinzen has received research grants from Medtronic, Boston Scientific, EBR Systems, Biological Delivery System Cordis, MSD and Proteus Medical, and is consultant for St. Jude Medical. Harry Crijns has received grant support from St. Jude Medical and Boston Scientific, and honoraria from Medtronic and BiosenseWebster. Kevin Vernooy has received research grants from Medtronic and St. Jude Medical and is consultant for Medtronic. The other authors declare no conflict of interest.
